# Development of the Weight-Related Sign and Symptom Measure

**DOI:** 10.1186/s41687-018-0042-9

**Published:** 2018-04-02

**Authors:** Meryl Brod, Lise Højbjerre, Kathryn M. Pfeiffer, Robyn Sayner, Henrik H. Meincke, Donald L. Patrick

**Affiliations:** 1 The Brod Group, 219 Julia Ave., Mill Valley, CA 94941 USA; 2 Novo Nordisk A/S, Vandtårnsvej 114, DK-2860 Søborg, Denmark; 3 University of Washington, Box 357660, Seattle, WA 98195-7660 USA

**Keywords:** Obesity, Overweight, Weight-related signs and symptoms, Quality of life, Patient-reported outcomes

## Abstract

**Background:**

Overweight and obesity have been associated with physical and emotional signs & symptoms. Research has shown that modest weight loss can mitigate some symptoms in individuals with overweight or obesity. This study’s purpose was to conduct concept elicitation (CE) interviews to provide documented qualitative support for the development of the Weight-Related Sign and Symptom Measure (WRSSM) to assess weight-related signs/symptoms in U.S. adults with overweight or obesity, with or without type 2 diabetes (T2DM).

Eight focus groups were conducted in the U.S. with adults with overweight or obesity to understand weight-related sign/symptom impact from the patient perspective. Individual interviews were conducted with clinical experts to understand the impact of overweight or obesity on patient signs and symptoms. Transcripts were analyzed to identify symptoms and observable signs. A clinical challenge was conducted with clinical experts to confirm the signs/symptoms were clinically relevant, important to patients, and would improve with modest weight loss. Cognitive debriefing (CD) was conducted with individuals with overweight or obesity to confirm readability and symptom relevance.

**Results:**

CE interviews were conducted with four clinical experts, and 61 people, 32% of whom had T2DM, participated in the focus groups. Analyses identified two major areas of obesity impacts: weight-related physical signs/symptoms, and emotional impacts. The most frequently reported physical signs/symptoms were feeling tired (74%), shortness of breath (69%), and joint pain (64%). The most often reported emotional impacts included poor self-image (72%) and depression (51%). Twelve signs/symptoms were identified during item generation and included on the preliminary measure. Twelve adults with overweight/obesity, who were not part of the focus groups, participated in CD. After the CD, a validation-ready, 10-item WRSSM measure was generated.

**Conclusions:**

Findings provide evidence of content validity for the validation-ready WRSSM in U.S. adults with overweight or obesity, including people with and without T2DM.

## Background

Over the last decade, the prevalence of obesity in adults has increased in the United States (U.S.) and worldwide [1, 2]. In the U.S., approximately 34% of the adult population has overweight (body mass index (BMI) of ≥25 and < 30) and 35% has obesity (BMI > 30) [1]. Worldwide, there are more than 1.9 billion adults with overweight or obesity [2].

Having overweight or obesity has been associated with increased morbidities including type 2 diabetes (T2DM), hypertension, cardiovascular disease, and stroke together leading to increased mortality [3, 4] and an increased risk of developing osteoarthritis [3]. Similarly, T2DM has been associated with an increased risk of macrovascular and microvascular complications [5–7]. Almost 90% of people living with T2DM have overweight or obesity [8]. Additionally, overweight and obesity have been associated with a reduction in health-related quality of life (HRQoL), as well as depression and feelings of anxiousness [3, 9–15].

Further, the prevalence of many of these comorbid conditions, such as hypertension, hypercholesteremia, coronary artery disease, heart failure, and stroke, has been shown to increase with each increasing weight category based on BMI, from overweight through Obese Classes I, II, and III [14, 16–18]. Individuals in increasing weight categories are also significantly more likely to report having chronic insomnia, fatigue, or lack of energy, osteoarthritis, neck, back, and joint pain, and gastroesophageal reflux disease [9, 17]. Moreover, sleep problems, low sexual desire, and dyspnea are experienced more often in individuals with obesity as compared to normal weight individuals [9, 19].

For individuals with overweight or obesity, weight loss has proven to be beneficial in reducing the morbidity and mortality from major cardiovascular events, hypertension, heart failure, and coronary heart disease [20, 21]. Weight loss has also been associated with a reduction in the prevalence of T2DM [4, 22]. Improvements in both chronic pain [23–26] and gastroesophageal reflux disease [27] are seen with weight loss. Several studies have shown the benefits of weight loss in individuals with T2DM, such as improvements in physical functioning and HRQoL, as well as reducing the risk of depression [28, 29].

The 2013 American Heart Association/American College of Cardiology/The Obesity Society obesity guidelines and the 2016 American Association of Clinical Endocrinologists & American College of Endocrinology guidelines endorse the benefits of weight loss; however, the guidelines do not specify whether weight loss would improve HRQoL [30, 31]. There are currently several reliable and responsive obesity-specific, patient-reported outcome (PRO) measures that have been developed and validated to assess the impact of being overweight or having obesity on HRQoL, such as the Impact of Weight on Quality of Life (IWQOL), IWQOL-Lite, Obesity and Weight-Loss Quality of Life (OWLQOL), and Impact of Weight on Activities of Daily Living (IWADL) [32–35]. However, these measures evaluate the function or behavior of an individual rather than the physical signs and symptoms experienced.

Currently, the Weight-Related Symptom Measure (WRSM) is the only PRO measure that assesses the symptoms that are commonly associated with obesity [35, 36]. There is evidence of validity for the WRSM based on psychometric analyses of data from four different studies that included individuals with obesity (BMI > 30) with and without comorbidities, as well as in individuals with overweight (BMI range, 27.0–29.9) plus one of the following comorbidities: hypertension, high cholesterol, or T2DM [36]. There is no evidence of validity for the WRSM measure in individuals with overweight, but *without* hypertension, high cholesterol, and/or T2DM. Moreover, the WRSM focuses on *symptoms*, but does not include *signs* of overweight/obesity. Additionally, the development of the WRSM measure did not adequately address possible overlap in symptoms that may be associated with both overweight/obesity and T2DM (e.g., fatigue or frequent urination). Given the frequent cooccurrence of overweight/obesity and T2DM, it is important to ensure that the measure includes symptoms that can be attributed to overweight/obesity rather than T2DM.

The purpose of this study was to develop a new measure of both the signs and symptoms of overweight and obesity in a diverse population of U.S. adults with overweight or obesity, including those with and without T2DM. In the development of the new measure, based in part on the WRSM measure, the study also aimed to provide evidence for the content validity of items in the measure based on rigorous qualitative research methodologies used for PRO measure development. Additionally, the study aimed to develop a preliminary theoretical model to identify relationships among key concepts, impacts, and modifiers related to the new PRO measure and to inform future studies using the measure.

## Methods

The study used standard methodologies for PRO measure development, in alignment with best research practices and U.S. Food and Drug Administration guidance on PRO development [37–41]. The qualitative analysis of concept elicitation interviews with clinical experts and focus group interviews with people with overweight or obesity was used to inform the structure and content of the measure and to develop the preliminary theoretical model. Once items were developed, a clinical challenge was conducted with clinical experts to ensure the relevance and importance of items in the measure, as well as the potential for change with weight loss. Finally, cognitive interviews were conducted with people with overweight or obesity to ensure that instructions and items were clear, relevant, and inoffensive, that recall period was appropriate, and that response options and scales were easily understood and appropriate.

### Concept elicitation

Concept Elicitation interviews and focus groups were conducted to identify key concepts that were both relevant and important to the target population to inform the development of the PRO measure [39]. The key concepts of interest were the signs and symptoms of overweight or obesity. A review of the current literature on overweight and obesity was used to inform the development of the semi-structured interview guides, which including open-ended questions and probes. For concept elicitation, individual interviews were conducted with clinical experts in obesity, and focus group interviews were conducted with adults with overweight or obesity.

Expert interviews were conducted by telephone with U.S. physicians following a semi-structured interview guide that asked what physicians considered to be important physical and emotional signs/symptoms of obesity, whether these signs/symptoms would improve with weight loss, how having T2DM would affect these signs and symptoms, and whether the signs/symptoms of obesity can be differentiated from signs/symptoms of T2DM in patients. Physicians were eligible if they: 1) were American Board of Obesity Medicine (ABOM) certified; 2) have been practicing for 2 or more years as a weight-loss physician; 3) spend at least 50% of their time caring for patients in a clinical setting; and 4) treat an average of 20 or more patients with overweight or obesity per month. Physicians were recruited and screened by a professional research organization using a proprietary database of physicians. The recruitment strategy ensured that physicians of differing backgrounds would be included (e.g. years of experience, gender, and geographic location). The telephone interviews lasted approximately 1 h and were recorded and transcribed.

Qualitative interviewing of participants in the target population is central to ensuring content validity in the development of PRO measures [38]. Focus groups interviews were chosen as a suitable method to provide a rich source of data on patient experiences from a broad range of adults in the target population with differing backgrounds and perspectives [39]. Eight focus groups were held in three U.S. cities in different regions. Focus groups were conducted separately by gender in each of the following overweight/obese class categories based on body mass index (BMI): Overweight (BMI range, 27.0–29.9); Obese Class I (BMI range, 30.0–34.9); Obese Class II (BMI range, 35.0–39.9), and Obese Class III (BMI ≥ 40). It should be noted that for the Overweight category, the BMI range of 27.0–29.9 was chosen to match the sample of an upcoming clinical trial in which the measure will be used, so it does not reflect the full BMI range typically used for Overweight classification. Respondents were eligible if they: 1) were 18 years or older; 2) self-reported a BMI from 27.0 to 29.9 for an Overweight focus group, BMI 30.0–34.9 for an Obese Class I (OC1) focus group, 35.0–39.9 for an Obese Class II (OC2) focus group, or BMI ≥ 40 for an Obese Class III (OC3) focus group; and 3) have not ever undergone surgery in order to lose weight. The exclusion criteria were having an eating disorder or having a cognitive impairment or other mental condition, including psychiatric disorders, that would impact one’s ability to participate in a focus group about obesity. The screener used by the professional research organization ensured that at least 25% of participants across weight classes self-reported a diagnosis of T2DM.

Respondents were recruited by a professional research organization that recruits for and hosts focus groups at their facilities. Informed consent was obtained from all participants. The study was approved by the Copernicus Group Independent Review Board in Durham, North Carolina.

All focus groups were conducted in-person at focus group facilities following a semi-structured focus group discussion guide, which was designed to elicit participants’ experiences of physical and emotional impacts related to their weight through the use of open-ended questions. The focus group discussion guide asked participants about what they considered to be physical and emotional signs/symptoms related to their weight and whether these would improve with weight loss. Respondents with T2DM were asked whether they could distinguish the signs/symptoms related to weight from those of T2DM. Each focus group was led by trained individuals with backgrounds in qualitative interviewing. Focus groups were conducted in an open-ended, conversational style, generally following the guide, but also responding to participants’ thoughts. Thus, while the focus groups were not identical, each followed the general themes and scope of the guide. The focus groups lasted approximately 2 h and were recorded and transcribed. Additionally, at the end of each focus group, participants completed a brief survey to report their experience of weight-related signs and symptoms and to rank how bothersome these signs and symptoms were. The main purpose of the brief survey was to give focus group participants an opportunity to report on experiences of weight-related signs and symptoms that they may not have felt comfortable discussing with the group. The survey included a list of symptoms from the original WRSM, as well as other signs and symptoms based on the literature review and expert knowledge. Participants also had the opportunity to report signs or symptoms that were not listed.

Transcripts were analyzed for content by conceptual themes using an iterative process [37]. Dedoose (www.dedoose.com), a web-based application for analyzing qualitative research data, was used for the qualitative data analysis. A preliminary code list of key concepts was developed based on the discussion guide. Transcripts were coded in chronological order, and emerging concepts were added to the coding scheme as they arose. Earlier transcripts were then checked for the new concepts. Each transcript was skimmed once, coded, and reviewed by two coders. Both coders conferred continuously to ensure consistency in coding. The codes were organized into two major concepts: physical signs and symptoms of overweight/obesity and weight-related emotional impacts.

### Item generation

The study team discussed the findings from the qualitative analysis to determine the rules of inclusion of signs and symptoms based on study participant and physician endorsement and to define the major and minor symptoms.

During 2 days of in-person meetings, the study team carefully considered and discussed the criteria for identifying signs and symptoms as major, and thus potential candidates for inclusion in the measure. In accordance with FDA guidelines and good practices for PRO measure development, the team sought to establish criteria to identify signs and symptoms that were important and relevant to patients, including patients with differing levels of severity of overweight or obesity and patients in differing demographic groups, and would be likely to change with weight loss, and would not be confused with another health condition [37–41]. Minimum percentages were included for participant and clinician endorsement of items, with some exceptions, to ensure the relevance of items to the target population and to avoid floor effects in the measure. The final agreed upon criteria for consideration of signs and symptoms as major included:

1. participant endorsement of at least 15% or for those between 10 and 14%, discussed and included if considered important conceptually;
2. clinician endorsement of at least 25%, unless it was a sign or symptom that they may not know about (e.g., sexual functioning);
3. the sign or symptom was rated as being very important or extremely important to participants in general;
4. the sign or symptom was reported across all four weight groups;
5. the sign or symptom was rated as being very important or extremely important to participants across both genders;
6. at least 15% of participants who experienced the sign/symptom reported that it improved with weight loss;
7. at least 25% of clinicians reported that the sign or symptom would improve with weight loss, unless it was a sign or symptom that they may not know about; and
8. the sign or symptom could not easily be confused with another health condition.

Minor signs or symptoms included all other signs or symptoms mentioned by the participants in the focus group discussions that were not classified as major.

After identifying major and minor signs and symptoms, the study team met to develop a first draft of the preliminary theoretical model. The purpose of the theoretical model was to hypothesize the relationships among the key concepts and impacts of interest, and to identify potential modifying factors [37]. The theoretical model also helped identify potentially confounding factors that need to be considered when generating items for the PRO measure and in future studies using the PRO measure. Based on the qualitative analysis, the major signs and symptoms identified, and the preliminary theoretical model, the team then generated the preliminary items to include in the measure and created an item definition table using the language of the participants as closely as possible for each item.

### Clinical challenge

Once the preliminary items were generated, a “clinical challenge” conference call was held with three additional physicians who were not included in the concept elicitation interviews to confirm that these were clinically relevant, important to patients, and could potentially improve with at least a modest weight loss (e.g., 5–10%) to ensure that items would be sensitive to change and appropriate for use in a clinical trial. The clinical challenge conference call lasted approximately 1 h.

To be eligible for the clinical challenge conference call and ensure that the physicians were experts in treating individuals with overweight or obesity, eligibility criteria included: 1) practicing medicine for a minimum of 10 years; 2) ABOM certification; 3) care for adult patients; 4) have a minimum of 50% of patients with overweight or obesity in their practice; and 5) have a minimum of 50% T2DM patients in their practice. The latter two were requirements as the clinicians needed to be able to answer questions about whether certain symptoms could be distinguished as being due to overweight/obesity or diabetes. Physicians were recruited and screened using a professional research organization.

### Cognitive debriefing interviews

Cognitive debriefing interviews were conducted to reach consensus on the appropriate format and structure of the measure, to ensure that items, response options and instructions were clear and relevant, to confirm that the recall period was reasonable, and to ensure that the content was comprehensive [37, 39].

The cognitive interviews were conducted in an independent sample of adults with overweight or obesity who met the same criteria as the focus group sample. They were recruited and screened by a professional research organization who also verified that they did not participate in the focus groups. Participants were emailed an informed consent form and the Weight Related Sign and Symptom Measure (WRSSM) in advance by the recruiter and were instructed to complete the survey 24–48 h prior to their scheduled individual telephone interview, to have the completed survey with them for the call, and to dial into a toll-free number at the scheduled time for the interview. A semi-structured interview guide with relevant probes was used to direct the cognitive interview process. Interview questions focused primarily on participants’ comprehension and perceptions of each item and the instructions, and also addressed issues of formatting, wording, content, recall period, response options, and relevance, using verbal probing techniques as needed.

The cognitive debriefing interviews were conducted individually by telephone in blocks of four participants each. After the first four participants were interviewed, the findings were reviewed, and a decision was made about what changes were needed to the measure. This process continued in blocks of four participants until a determination was made that the readability and relevance were acceptable based on consensus agreement among the participants in the block. At the end of cognitive debriefing, a validation-ready version of the measure was produced, and the preliminary theoretical model was updated to incorporate findings from the clinical challenge and the cognitive debriefing interviews.

## Results

### Concept elicitation

#### Sample description

A total of four expert interviews were conducted with U.S. physicians, all of whom were board certified in Obesity Medicine. On average, the physicians were in practice for 15 years (range: 10–22 years) and in Obesity Medicine for 14 years (range: 9–22 years). Physicians reported seeing an average of 173 obesity patients per month (range: 40–300 patients). Three of the four physicians reported working in a private practice/outpatient clinic, and one worked at an academic teaching hospital. Three physicians reported an academic affiliation.

Sixty-one people participated in the eight focus groups. The focus groups for Overweight, OC1, and OC3 weight classes each had eight men and eight women. In the OC2 focus groups, there were five men and eight women. Participants reported varying household income levels and were diverse in terms of racial/ethnic background.

The focus group sample was 53% female, and the average age was 51.5 years. The average number of self-reported comorbid conditions per patient was 3.3 (*n* = 57). The self-reported comorbid conditions reported most frequently were hypertension (*n* = 32, 56.1%), T2DM (*n* = 20, 35.1%), sleep apnea (*n* = 19, 33.3%), and reflux diseases (*n* = 14, 24.6%). The average number of comorbidities per patient by weight class was Overweight: 3.3 (range: 0–10); OC1: 3.4 (range: 1–7); OC2: 3.4 (range 1–6); and OC3: 2.9 (range: 0–10). Table 1 presents the demographics and health characteristics of the study participants.

**Table 1:** Focus group participant demographic and health characteristics

Demographic characteristics	Totals (*n* = 61)
Age, average (range)	51.5 (27–73)
Gender, n (%)	
Female	32 (52.5)
Male	29 (47.5)
Race/ethnicity, n (%)	
Asian	8 (13.1)
Black/African American	11 (18.0)
Hispanic/Chicano/Latino	10 (16.4)
“White”/Caucasian	30 (49.2)
Other / Prefer not to answer	2 (3.3)
Household Income, n (%)	
Less than $25,000	8 (13.1)
$25,000–$49,999	12 (19.7)
$50,000–$74,999	12 (19.7)
$75,000–$99,999	15 (24.6)
$100,000–$149,999	9 (14.8)
$150,000 or more	5 (8.2)
BMI Focus Group Participants, n (%)	
Overweight (27.0–29.9)	16 (26.2)
Obese Class I (30.0–34.9)	16 (26.2)
Obese Class II (35.0–39.9)	13 (21.3)
Obese Class III (≥ 40)	16 (26.2)
Told by doctor have weight problem, n (%)	52 (85.2)
Duration (years) of having obesity, average (range)	17.5 (1–44)
Self-Reported Other health conditions^a^, n (%)	
Asthma	8 (14.0)
Cardiovascular disease	5 (8.8)
Chronic back pain	13 (22.8)
Depression	3 (5.3)
Diabetes (type 2 diabetes)	20 (35.1)
Dyslipidemia	15 (26.3)
Gallstones or gall bladder disease	6 (10.5)
Gynecological problems	3 (5.3)
Hypertension	32 (56.1)
Infertility	3 (5.3)
Metabolic syndrome	2 (3.5)
Obesity	28 (49.1)
Osteoarthritis	11 (19.3)
Reflux diseases	14 (24.6)
Sleep apnea	19 (33.3)
Stomach and intestinal problems	5 (8.8)
Stroke	1 (1.8)
Other	12 (21.1)

^a^*n* = 57 since four people declined to answer

Three physicians participated in the clinical challenge interview. On average, the physicians were in practice for 14 years (range: 10–20 years). They reported having an average of 70% of patients with overweight or obesity (range: 60–75%) and an average of 75% of patients with diabetes (range: 65–85%) in their clinical practice site.

### Qualitative analysis

Thematic saturation was assessed by creating a table organized by concept codes and focus groups listed in the order in which they occurred to track when new concepts appeared. After Focus Group 2, 71% of all concepts had been discussed, and by Focus Group 6, 95% of concepts were covered. Total saturation of concepts was reached after the sixth focus group.

Participants described 35 physical signs and symptoms during the focus group discussions. Table 2 presents these signs and symptoms by weight class and gender. The physical signs and symptoms reported by at least 15% of all participants included feeling tired (*n* = 45, 74%), shortness of breath (*n* = 42, 69%), joint pain (*n* = 39, 64%), back pain (*n* = 33, 54%), low physical stamina (*n* = 32, 52%), trouble sleeping (*n* = 23, 38%), sensitivity to heat (*n* = 21, 34%), low energy (*n* = 19, 31%), snoring (*n* = 14, 23%), general discomfort (*n* = 13, 21%), acid reflux (*n* = 12, 20%), increased thirst (*n* = 12, 20%), heavy sweating (*n* = 11, 18%), and frequent urination (*n* = 10, 16%).

**Table 2:** Physical signs and symptoms by gender and weight class

*Sign/symptom experienced* *n (%)*	Gender		Overweight/Obesity Class			
	Men *n* = 29	Women *n* = 32	Overweight *n* = 16	Obese Class I *n* = 16	Obese Class II *n* = 13	Obese Class III *n* = 16
Feeling tired	20 (69)	25 (78)	13 (81)	11 (69)	11 (85)	10 (63)
Shortness of breath	17 (59)	25 (78)	10 (63)	11 (69)	10 (77)	11 (69)
Joint pain	16 (55)	23 (72)	9 (56)	11 (69)	8 (62)	11 (69)
Back pain	15 (52)	18 (56)	5 (31)	10 (63)	10 (77)	8 (50)
Low physical stamina	13 (45)	19 (59)	10 (63)	10 (63)	10 (77)	2 (13)
Trouble sleeping	13 (45)	10 (31)	5 (31)	5 (31)	4 (31)	9 (56)
Sensitivity to heat	9 (31)	12 (38)	5 (31)	3 (19)	11 (85)	2 (13)
Low energy	6 (21)	13 (41)	2 (13)	5 (31)	8 (62)	4 (25)
Snoring	10 (34)	4 (13)	3 (19)	3 (19)	3 (23)	5 (31)
General body discomfort	8 (28)	5 (16)	4 (25)	3 (19)	2 (15)	4 (25)
Acid reflux	1 (3)	11 (34)	3 (19)	2 (13)	5 (38)	2 (13)
Increased thirst	7 (24)	5 (16)	4 (25)	2 (13)	2 (15)	4 (25)
Heavy sweating	5 (17)	6 (19)	2 (13)	2 (13)	6 (46)	1 (6)
Frequent urination	7 (24)	3 (9)	4 (25)	3 (19)	1 (8)	2 (13)
Foot pain	4 (14)	4 (13)	0 (0)	4 (25)	2 (15)	2 (13)
Muscle aches	5 (17)	3 (9)	2 (13)	2 (13)	2 (15)	2 (13)
Protruding abdomen	3 (10)	5 (16)	3 (19)	5 (31)	0 (0)	0 (0)
Low sexual desire	3 (10)	5 (16)	2 (13)	2 (13)	1 (8)	3 (19)
Swelling/inflammation	3 (10)	4 (13)	0 (0)	4 (25)	1 (8)	2 (13)
Lightheadedness	7 (24)	0 (0)	3 (19)	4 (25)	0 (0)	0 (0)
Water retention	2 (7)	4 (13)	3 (19)	0 (0)	2 (15)	1 (6)
Balance problems	5 (17)	0 (0)	1 (6)	3 (19)	1 (8)	0 (0)
Skin irritation	2 (7)	3 (9)	1 (6)	4 (25)	0 (0)	0 (0)
Urine leakage	1 (3)	3 (9)	0 (0)	3 (19)	1 (8)	0 (0)
Immune system	3 (10)	0 (0)	0 (0)	0 (0)	0 (0)	3 (19)
Increased appetite	1 (3)	2 (6)	2 (13)	0 (0)	0 (0)	1 (6)
Injury prone	2 (7)	1 (3)	1 (6)	2 (13)	0 (0)	0 (0)
Sexual functioning	0 (0)	3 (9)	0 (0)	0 (0)	2 (15)	1 (6)
Headaches	2 (7)	0 (0)	0 (0)	2 (13)	0 (0)	0 (0)
Hormonal issues	0 (0)	2 (6)	0 (0)	0 (0)	0 (0)	2 (13)
Sensitivity to cold	0 (0)	2 (6)	0 (0)	0 (0)	1 (8)	1 (6)
Blurry vision	1 (3)	0 (0)	0 (0)	0 (0)	0 (0)	1 (6)
Poor circulation	0 (0)	1 (3)	0 (0)	1 (6)	0 (0)	0 (0)
Tingling in limbs	0 (0)	1 (3)	0 (0)	0 (0)	0 (0)	1 (6)
Varicose/spider veins	0 (0)	1 (3)	1 (6)	0 (0)	0 (0)	0 (0)

Due to the qualitative nature and focus of the study, as well as the small sample size, statistical significance tests were not used to determine differences between or among groups. Thus, any reported group differences in reported frequencies of signs or symptoms should be interpreted with caution. Nevertheless, there were some observed differences by weight class, gender, and presence of T2DM that were relatively large and should be noted. Low physical stamina was reported most often by those in the Overweight, OC1, and OC2 weight classes (Overweight *n* = 10, 63%; OC1 *n* = 10, 63%; OC2 *n* = 10, 77%), but was reported by only two participants (13%) in the OC3 weight class. Sensitivity to heat was reported most often by OC2 weight class (*n* = 11, 85%) and in fewer proportions by those in the other weight classes (Overweight *n* = 5, 31%; OC1 *n* = 3, 19%; OC3 *n* = 2, 13%). Heavy sweating was reported most often by OC2 participants (*n* = 6, 46%) and less so in the other weight classes (Overweight *n* = 2, 13%; OC1 *n* = 2, 13%; OC3 *n* = 1, 6%).

The most frequently reported signs and symptoms reported by each gender included feeling tired (men *n* = 20, 69%; women *n* = 25, 78%), shortness of breath (men *n* = 17, 59%; women *n* = 25, 78%), joint pain (men *n* = 16, 55%; women *n* = 23, 72%), back pain (men *n* = 15, 52%; women *n* = 18, 56%), and low physical stamina (men *n* = 13, 45%; women *n* = 19, 59%). The physical signs and symptoms that were reported by at least 15% of all participants were reported in similar proportions across gender except low energy (men *n* = 6, 21%; women *n* = 13, 41%), acid reflux (men *n* = 1, 3%; women *n* = 11, 34%), and frequent urination (men *n* = 7, 24%; women *n* = 3, 9%).

The weight-related signs and symptoms were reported in similar proportions by those with and without T2DM with few exceptions, including heavy sweating (with T2DM *n* = 6, 30%; without T2DM *n* = 5, 12%), trouble sleeping (with T2DM *n* = 4, 20%; without T2DM *n* = 19, 46%), and low sexual desire (with T2DM *n* = 1, 5%; without T2DM *n* = 7, 17%).

Participants described seven emotional impacts related to weight during the focus group discussions. Table 3 displays the weight-related emotional impacts by weight class and gender. The weight-related emotional impacts reported by at least 15% of all participants included poor self-image (*n* = 44, 72%), depression (*n* = 31, 51%), irritability (*n* = 15, 25%), and isolation (*n* = 10, 16%).

**Table 3:** Weight-related emotional symptoms by gender and weight class

*Emotional impact* *n (%)*	Gender		Overweight/Obesity Class			
	Men *n* = 29	Women *n* = 32	Overweight *n* = 16	Obese Class I *n* = 16	Obese Class II *n* = 13	Obese Class III *n* = 16
Poor self-image	19 (66)	25 (78)	13 (81)	12 (75)	11 (85)	8 (50)
Depression	11 (38)	20 (63)	9 (56)	6 (38)	7 (54)	9 (56)
Irritability	4 (14)	11 (34)	5 (31)	3 (19)	5 (38)	2 (13)
Isolation	6 (21)	4 (13)	1 (6)	5 (31)	4 (31)	0 (0)
Anger	4 (14)	2 (6)	2 (13)	2 (13)	0 (0)	2 (13)
Frustration	3 (10)	3 (9)	1 (6)	1 (6)	2 (15)	2 (13)
Worry	4 (14)	1 (3)	0 (0)	3 (19)	1 (8)	1 (6)

The most frequently reported emotional impacts reported across all weight classes were poor self-image (Overweight *n* = 13, 81%; OC1 *n* = 12, 75%; OC2 *n* = 11, 85%; OC3 *n* = 8, 50%), depression (Overweight *n* = 9, 56%; OC1 *n* = 6, 38%; OC2 *n* = 7, 54%; OC3 *n* = 9, 56%), and irritability (Overweight *n* = 5, 31%; OC1 *n* = 3, 19%; OC2 *n* = 5, 38%; OC3 *n* = 2, 13%). There were some differences in the proportions of emotional impacts reported by each weight group. Isolation was reported most often by OC1 (*n* = 5, 31%) and OC2 (*n* = 4, 31%) weight classes and by only one Overweight (6%) and no OC3 participants.

The emotional impacts most frequently reported across both genders include poor self-image (men *n* = 19, 66%; women *n* = 25, 78%) and depression (men *n* = 11, 38%; women *n* = 20, 63%). Eleven women (34%) reported irritability as compared to only four men (14%). Men reported isolation (*n* = 6, 21%) more often in the focus group discussions than women (*n* = 4, 13%).

### Preliminary theoretical model

**Fig. 1:**
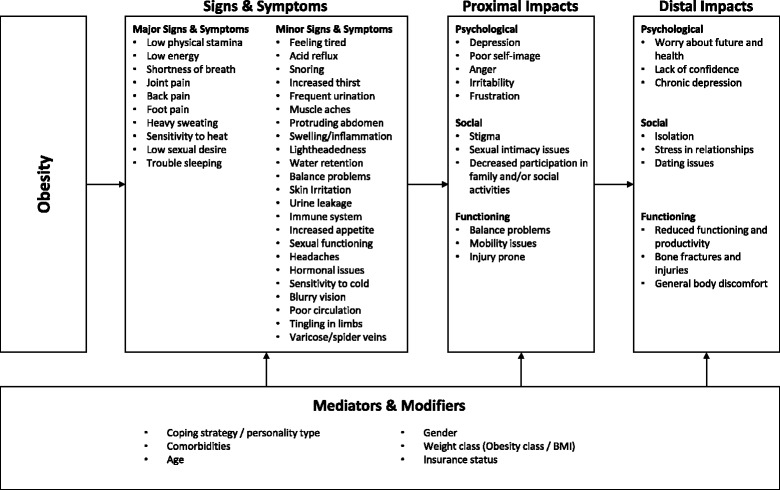
Theoretical Model of the Weight-Related Sign and Symptom Measure

Based on the qualitative analysis of the concept elicitation interviews and focus groups, a preliminary theoretical model of how overweight/obesity affects patients’ functioning and well-being was developed (Fig. 1). The model illustrates the hypothesized relationships among the major and minor physical signs and symptoms, proximal impacts, and distal impacts along with potential mediators and modifiers. Proximal impacts were considered more immediate, while distal impacts were considered longer-term. Although emotional issues were important to many of the respondents with overweight or obesity, the study team considered emotional issues to be psychological impacts rather than part of the target concepts for the measure, the physical signs and symptoms of overweight or obesity. Additionally, it is often difficult to assign attribution for psychological factors to any one cause. Potential modifiers and mediators, which may mitigate or amplify the impact of overweight or obesity in individuals, include coping strategy/personality type, comorbidities, age, gender, and weight class. The model is considered preliminary in nature, as it may require modification based on future research.

### Item generation

The purpose of the measure is to assess the major, clinically meaningful signs and symptoms of having overweight or obesity. Thus, items were generated only for the major signs and symptoms while minor signs and symptoms, as well as proximal or distal impacts, were excluded from the measure. Based on the criteria outlined above, 12 signs and symptoms were categorized as major, including: low physical stamina, shortness of breath, joint pain, back pain, foot pain, trouble sleeping, sensitivity to heat, heavy sweating, low energy, feeling tired, low sexual desire, and poor sexual functioning.

### Clinical challenge

Based on the clinical challenge, the final list of sign and symptom items was generated, and the preliminary draft of the WRSSM measure was developed. All items that had at least two of the three clinical challenge physicians in agreement that the item was 1) a major sign or symptom of obesity and 2) frequently reported by their patients were included in the preliminary WRSSM measure that went through cognitive debriefing. Poor sexual functioning was excluded from the preliminary measure since it did not meet the inclusion criteria. Acid reflux was added to the preliminary measure since two of the three physicians agreed that it was a major sign/symptom of obesity and that their patients frequently reported it.

### Cognitive debriefing

A total of 12 adults participated in cognitive debriefing interviews. Each block of four participants was stratified by weight class (Overweight, OC1, OC2, OC3) and screened to include one person with diabetes per block. Half were female, the average age was 47 years and the majority were working for pay (91.7%). The average number of self-reported comorbid conditions per participant was 3.1, and average BMI was 35.6.

After the first block of interviews, findings were reviewed, and a decision was made as to whether any changes to the WRSSM measure were necessary. This process continued in blocks of four participants until a determination was made that the readability, clarity and relevance of all instructions and items, including response options and response scale format, were acceptable based on consensus agreements among respondents in an entire block. A total of three blocks were necessary to refine the WRSSM measure items in terms of readability and relevance. An additional four interviews were conducted with participants from previous blocks to refine the instructions only. These brief, confirmatory interviews resulted in acceptance by the entire block of minor changes to the instructions.

The cognitive debriefing resulted in the validation-ready Weight Related Sign and Symptom Measure, named the WRSSM (WRSM V2), which is made up of 10 items using the same stem and response options. For example, respondents are asked, “During the past 7 days, because of your weight, how often did you experience joint pain (in any part of your body)?” Response options were based on a 5-point Likert-type scale, which was chosen to ensure that meaningful distinctions could be made among responses for analysis while minimizing the cognitive burden for respondents completing the instrument [42]. Response options included the following: “Never/almost never,” “Rarely,” “Sometimes,” “Often,” and “Always/almost always.” A rating scale, which is frequently used to measure patient-reported health status and symptom severity, was used to measure the severity of respondents’ signs or symptoms [43]. Each item includes a sub-item measuring sign/symptom severity among those who experienced the sign/symptom in the past 7 days using a rating scale ranging from 0 to 10, where 0 indicates “not bad at all,” and 10 indicates “worst imaginable.” For instance, for joint pain, respondents are asked, “If you experienced joint pain, how bad was it at its worst?” Respondents are then instructed to circle a number on the rating scale, which is consistent across sub-items, to indicate the sign/symptom severity they experienced in the past 7 days. The 10 signs or symptoms included in the updated WRSSM measure include shortness of breath, joint pain, low physical stamina, back pain, low energy, sensitivity to heat, trouble sleeping, heavy sweating, low sexual desire, and foot pain.

The WRSSM (WRSM V2) differs from the original WRSM in that 11 items have been dropped. Skin irritation, urine leakage, increased appetite, sensitivity to cold, increased irritability, and lightheadedness were excluded from the WRSSM because these items did not fit the inclusion criteria for the updated measure, as less than 10% of the focus group participants reported these as being important weight-related physical signs or symptoms. Also, snoring, increased thirst, frequent urination, and water retention were excluded from the updated measure since these did not meet the inclusion criteria. Lastly, feeling tired was dropped from the updated measure during cognitive debriefing because patients were not able to distinguish whether this was related to their weight or other medical conditions. Additionally, a new item, low energy is included in the WRSSM since it was reported as important to patients, clinically relevant and would improve with weight loss.

Table 4 presents representative quotes for each of the 10 WRSSM (WRSM V2) items.

**Table 4:** Selected Quotes for Major Signs and Symptoms

	Selected Quotes
Shortness of breath	*…you’re toting on this extra baggage and I find it’s very difficult to breathe sometimes. I get a lot of episodes where I feel like I’m short of breath or I just can’t breathe as relaxed as I want to breathe. (Overweight female)* *When I was at my biggest weight, I was out of breath even going upstairs. Now I’m going up and down the stairs and I don’t have that issue now, so I can definitely see the difference. (Obese Class III male)*
Joint pain	*For me it’s the ankles…I notice what really hurts the most, worse than my knees, and I have arthritis in the knees, is the ankles. The ankles are really taking a pounding holding up this much weight. (Obese Class III female)* *I have achy joints…So knee problems, knee issues. I can’t stand for too long. I have to sit down. I can’t go up the stairs. (Obese Class I female)*
Back pain	*…when I gained some weight, it put more pressure on my back, so it aggravated my symptoms more. So, when I started to lose some weight, I could have a little more relief. (Overweight male)* *Yeah, it brings you to a slouch. When your gut sticks out, I mean, it just puts a strain on you…It hurts…it’s hard on your back. (Obese Class I male)*
Low physical stamina	*Just like pace yourself, things that would be routine, now you find yourself, I can do it for about 15 min, then okay, then rest, then go back to do it again. I used to be able to do it all at once…I think weight has something to do with that. Obviously, age also. (Obese Class I male)* *It makes it a little difficult that I have to plan my day around my activities, around my weight … but I do know that losing weight has helped. I have lost 15 pounds in the last 3 months, and I have noticed that has helped quite a bit, but it still makes it hard to keep up your stamina. (Obese Class II female)*
Trouble sleeping	*…there are times where I usually get four or 5 h of sleep, but it’s just constantly tossing and turning, from not being comfortable. Just being so big I guess I can’t get comfortable. When I was thinner, it was easier. I could lie down at any time and never hurt anything. (Obese Class II female)* *… I used to sleep in on the weekend but now with my son I don’t get to sleep in, so I never get to recover. And I think part of that is just because I have sleep apnea … because of my weight, and I can’t get a really good night’s sleep because of it. It’s probably been years. (Obese Class III male)*
Sensitivity to heat	*So, I am hot all of the time and cold…it is different when I am thinner. (Obese Class II female)* *My wife calls me the human furnace. Even in the wintertime I need a window open and a fan on because number one, I’m either hot and I can’t breathe. (Obese Class II male)*
Low energy	*I always had an extremely high energy level and the heavier I’ve gotten the more that my energy level has dramatically dropped…I have to constantly battle what part of me wants to stay home and rest and another part of me saying you’ve got to get up and go to work. A lot of it is my energy level. (Obese Class III female)* *I have more energy. I know that much because like I’ve said, lately I’ve been maintaining this weight. When I did lose some weight, I was able to do more things. I wasn’t always taking a deep breath. Like doing the laundry, I’ll do a load of laundry and have to bend down, pick it up, put it in the machine. By the time I’m done with that, I’m like, okay. I need a break. (Obese Class II male)*
Heavy sweating	*I sweat like crazy. When I was heavier, I was just sitting in the shade by a tree. I’d be sweating…Now that I’ve lost a little weight it’s not as bad. (Obese Class III male)* *But I mean it’s still good to have a fan on because I am just overweight and when you have all that weight…your body just tends to increase the temperature especially when you sleep. For some reason, for me I have been sweating in my sleep, so I have to sleep with the fan on. (Obese Class II female)*
Foot pain	*Then I’ve also had issues with plantar fasciitis, which is, like, heel pain. And the podiatrist said it’s all the extra weight. For every step I take, it makes it really bad for my heels, so definitely extra weight causes problems. (Obese Class I female)* *Yes, I get foot pain sometimes. It feels like… At the top of my foot, not the bottom. Like the muscle feels pulled, stressed. (Obese Class III female)*
Low sexual desire	*So, the reason I have one kid is I just don’t have the stamina or just don’t have that drive. If you’re in good shape, you’re always…everything is working the way it should be. (Overweight male)*

## Discussion

These results suggest that the signs and symptoms of overweight/obesity, as well as their impacts, are multi-faceted and may vary by weight class and gender, as indicated in the preliminary theoretical model. Consistent with prior research, study participants frequently reported comorbidities that have previously been found to be associated with overweight or obesity, including hypertension and T2DM [3, 4]. Participants also described many different physical signs and symptoms, as well as emotional impacts, due to overweight or obesity that have been reported in other research [9, 17, 19]. Generally, the signs and symptoms were reported more frequently in each increasing weight category – from Overweight to OC1 to OC2, which is consistent with previous studies [9, 17]. Interestingly, fewer participants in the OC3 focus groups reported weight-related signs and symptoms as compared to those in the OC2 focus groups. This may be partially due to OC3 individuals adapting to the signs and symptoms as they may have had obesity for longer than those in the other weight-classes. Also, OC3 individuals may be less mobile and thus, may not report symptoms such as low physical stamina, sensitivity to heat, or having low energy. Additionally, OC3 individuals, on average, had fewer comorbidities than the other weight-classes.

Based on the composition of the concept elicitation sample, this study provides evidence for the content validity of the following items in the WRSSM in individuals with overweight without comorbidities, as well as for individuals with overweight or obesity with or without T2DM: shortness of breath, joint pain, back pain, low physical stamina, sensitivity to heat, heavy sweating, foot pain, and low sexual desire. However, the 10-item validation-ready WRSSM (WRSM 2.0) does not include several items that were included in the WRSM in addition to including a new item: low energy [36]. The differences between these measures may be due to the inclusion of both overweight individuals without comorbidities and at least one-fourth of all individuals having T2DM in the current study.

It should be noted that there were several emotional concerns attributed to overweight/obesity – poor self-image, depression, and irritability – that at least 25% of participants reported as being important. These emotional concerns were excluded from the WRSSM as these were considered to be proximal or distal impacts rather than major physical signs and symptoms of obesity. However, their major impact on the lives of individuals with overweight or obesity is well documented in prior research and should not be ignored [10, 11, 15].

As with all studies, there are some limitations to this study which should be taken into consideration when interpreting findings. The concept elicitation expert interviews and the clinical challenge interviews were conducted with physicians who had ABOM certification. Other healthcare providers, including nurses and physical therapists, may have differing perspectives. As is typical in focus group research, some participants spoke more than others, and not all participants answered every question. Nevertheless, the focus group facilitators ensured that all participants contributed to the discussion. Further, half of the focus group respondents reported being depressed about their weight, and this may have influenced how participants responded to the interviews. Also, there may be other important mediators or moderators not observed in the study, such as insurance status, that influence how people experience signs, symptoms, or impacts.

Further, there may be some limitations in the generalizability of results. Although focus group participants were diverse in terms of background, including race/ethnicity, gender, age, and socioeconomic status, results may differ for some groups who were excluded from the study. Specifically, respondents who had an eating disorder or who had a cognitive impairment or other mental condition, including psychiatric disorders, that would impact one’s ability to participate in a focus group about obesity, were excluded from the focus groups. Thus, the results may not be generalizable to these groups.

The study also suggests areas for future research. There may be differences in weight-related signs and symptoms by weight class group, gender, racial/ethnic background, which should be explored in future studies. Lastly, since the WRSSM was developed in the U.S. with English-speaking participants, cultural and linguistic equivalency should be examined for other countries.

## Conclusions

This study found that signs and symptoms of overweight and obesity are multifaceted and diverse. The identification of the major patient-reported signs and symptoms of overweight or obesity can provide the basis for a validated PRO measure that could allow clinicians and researchers to assess weight-loss treatments. Additional research is needed to validate the WRSSM in patients with overweight or obesity to determine whether it is a reliable measure of the impact of weight on patients with overweight or obesity, with or without T2DM. While the preliminary theoretical model may be used to inform future research using the WRSSM measure, modifications to the model may be required.

## Abbreviations

ABOM: American Board of Obesity Medicine
BMI: Body mass index
CD: Cognitive debriefing
CE: Concept elicitation
HRQoL: Health-related quality of life
IWADL: Impact of Weight on Activities of Daily Living
IWQOL: Impact of Weight on Quality of Life
OC1: Obese Class I
OC2: Obese Class II
OC3: Obese Class III
OWLQOL: Obesity and Weight-Loss Quality of Life
PRO: Patient-reported outcomes
T2DM: Type 2 diabetes
WRSM: Weight-Related Symptom measure
WRSSM: Weight Related Sign and Symptom measure (WRSM V2.0)
